# Saddle pressures distribution at different pedaling intensities in young off-road cyclists: focus on sex

**DOI:** 10.1080/07853890.2025.2495764

**Published:** 2025-04-29

**Authors:** Domenico Savio Salvatore Vicari, Antonino Patti, Valerio Giustino, Flavia Figlioli, Daniele Zangla, Nemanja Maksimovic, Patrik Drid, Antonio Palma, Antonino Bianco

**Affiliations:** a Sport and Exercise Sciences Research Unit, Department of Psychology, Educational Science and Human Movement, University of Palermo, Palermo, Italy; b Department of Neurosciences, Biomedicine and Movement Sciences, University of Verona, Verona, Italy; c PhD Program in Health Promotion and Cognitive Sciences, University of Palermo, Palermo, Italy; d Faculty of Sport and Physical Education, University of Novi Sad, Novi Sad, Serbia

**Keywords:** Bicycle, bike, cycling, cyclists, saddle pressures, biomechanics

## Abstract

**Background:**

The pressures on the saddle depend on several factors and can compress specific neurovascular tissues, leading to acute and chronic genital pathologies. Based on the pelvic differences between males and females, the aim of this study was to explore any differences on saddle pressures distribution according to sex.

**Methods:**

Twenty young off-road cyclists (10 m, 10 f) were recruited. Each participant was evaluated on own bike installed on a specific bike roller with a magnetic resistance. Firstly, each participant was asked to warm-up for 10 min at a self-selected cadence and intensity. Then, saddle pressures distribution was measured at three different pedaling intensities (100, 140, 180 W) with a pedaling cadence of 90 rpm, using a device equipped with sensors capable of acquiring pressures.

**Results:**

A significant difference in the ischial distance was found between males and females (*p* = 0.007). As pedaling intensity increased, results showed a significant higher pressure in the pubic region (*p* = 0.004) in males and a significant higher pressure in the posterior region in females (*p* = 0.034). *Post hoc* multiple comparisons test revealed a significant increase from 100 to 180 W (*p* = 0.003) in the pubic region pressure in males, while no significant differences were detected in the posterior region pressure in females.

**Conclusions:**

In male off-road cyclists, the pressure in the pubic region is higher with increasing pedaling intensity. Hence, to prevent acute and chronic genital pathologies, it would be advisable to fix the saddle in the best possible way during the bike fitting.

## Introduction

Bicycle riding is an aerobic non-impact exercise with protective effects and beneficial influence on the risk of hypertension, diabetes and stroke [1]. However, cycling training has also been shown to cause acute traumatic and overuse injuries [2,3].

While pedaling, cyclists distribute their body weight on the pedals, handlebars, and saddle. It would appear that the pressure on the saddle can compress specific neurovascular tissues leading to acute and chronic genital pathologies [4–7]. In particular, these may be genital numbness, erectile dysfunction, priapism, affecting serum prostate-specific antigen levels, hematuria and infertility [8]. The prevalence of these pathologies in the perineum range from 7 to 61% and the prevalence of erectile dysfunction from 4 to 19% [9]. Based on different existing studies, it has been reported that erectile dysfunction has a prevalence of 4% [10], 13% [11], 17% [12], 19% [13]. These pathologies show a higher frequency of occurrence in cyclists of mountain biking, also known as off-road cycling [9]. In fact, those who practice mountain biking travel on uneven paths and are, therefore, subject to continuous stresses on the saddle causing repeated microtraumas of the perineum which can lead to perineal folliculitis, subcutaneous boils, and nodules [14].

As a matter of fact, perineum pain is one of the most frequent complications that can lead to suspension of sporting activity by the athlete. The causes of pain are multifactorial and, in fact, the setting, mobility of the pelvis, type of saddle, and width of the saddle can contribute to the painful symptoms.

Some research groups explored potential preventive strategies and exercises to avoid excessive saddle pressures in cyclists [5,6].

The analysis of the pressures exerted on the saddle, based on the relationship between the force applied to a surface, represents the most suitable approach to study their magnitude and the contact surfaces, allowing the prevention of the risk of injuries [15]. It would appear that for an ideal saddle pressures distribution the ischial tuberosities should exert the highest pressures and that these should gradually decrease towards the pubic region [16]. As a consequence, an abnormal distribution of pressures on the saddle could stress delicate biological tissues, organs, and glands leading to acute and chronic genital pathologies [2].

The presence of a cut-out has been shown to influence pelvic tilt and perceived comfort among female cyclists [17]. Moreover, a previous study found that saddles with a partial cut-out or without a nose reduced perineal pressure in male cyclists [18]. Further studies have shown that saddle design can affect blood flow in the perineal region [19,20], which is another measure for evaluating comfort and causes saddle-related pathologies such as numbness [21,22] and erectile dysfunction [19,20,23–25].

Considering the pelvic anatomical differences between males and females, this study aimed to comprehensively explore the potential variances in saddle pressures distribution according to sex. Building upon preliminary findings that indicated possible sex differences in saddle pressures [26], we hypothesized a variation in the distribution of these pressures, especially given the greater width between the ischial tuberosities in females which might reduce the pressure in the pubic region.

## Materials and methods

### Study design

This is a cross-sectional study in which saddle pressures were measured at three different pedaling intensities in young off-road cyclists of both sexes.

### Participants

For participant enrollment, a member of the Research and Development Center of the Sicilian Regional Committee of the Italian Cycling Federation (FCI) contacted local cycling teams in the Sicily region (Italy) to present the research. Subsequently, it was presented to the cyclists and parents that had expressed their willingness to participate. The participation of the cyclists was voluntary and required a written informed consent from their parents, since the participants were minors.

To be eligible for the study, participants had to meet the following inclusion criteria: (a) to be aged between 10 and 12 years (Italian youth categories: G4, G5, G6); (b) to practice mountain biking for at least 1 year; (c) to practice mountain biking at least 3 h/week. The exclusion criteria were the following: (a) no musculoskeletal injuries in the previous 6 months; (b) no history of saddle sores, skin irritations in the perineal region, perineal nodules, or perineal numbness.

Hence, twenty young off-road cyclists (*m* = 10; *f* = 10) were recruited. Participants’ characteristics are shown in Table 1.

**Table 1.** Characteristics of the participants.

	Sample (mean ± SD)	*m* (mean ± SD)	*f* (mean *±* SD)
*n*	20	10	10
Age (years)	10.45 ± 1	10.8 ± 1.14	10.1 ± 0.74
Height (cm)	147.65 ± 8.04	150.1 ± 10.13	145.2 ± 4.54
Weight (kg)	44.6 ± 6.32	46.1 ± 8.09	43.1 ± 3.73

m: male; f: female; SD: standard deviation.

The study, in accordance with the principles of the Declaration of Helsinki for the use of people in research, was approved by the Bioethics Committee of the University of Palermo (n. 132/2023).

### Procedures

All procedures were carried out during the summer transitional period (i.e. in the absence of any competition) by the same investigator and in the same time slot at the laboratory of the Sport and Exercise Sciences Research Unit of the University of Palermo.

### Bike fitting

Two weeks prior to data collection, in order to standardize procedure measurements, each participant was given a bike fitting to optimize joints’ function.

For the bike fitting, a spirit level was used to fix the top surface of the saddle in a horizontal position. Nine markers were placed at specific anatomical points for each participant (ulnar styloid, humeral epicondyle, acromion, iliac wing, greater trochanter, iliotibial hemirima, lateral malleolus, head of the 5th metatarsal, central axis of the pedal). Next, the shoe adjustment was carried out [27].

Then, an action camera (GoPro 9, GoPro Inc., San Mateo, CA, USA) set at an acquisition rate of 60 fps (frames per second) was positioned horizontally with respect to the ground with a suitable support and at a distance of 2 meters from the bike. Once the video recording started, each participant was asked to pedal for 60 s at an intensity of 140 W while maintaining a pedaling cadence of 90 rpm (revolutions per minute).

Subsequently, from the video acquisition, a federal technician performed the bike fitting for each participant in which mechanical modifications to the bike were made in order to achieve the following joint ranges of motion: knee flexion angle of 30°–40° [28,29], plantar flexion angle of 15°–30° [30], upper limb bending angle of 150°–170° [30]. Regarding the trunk flexion angle, since no references are presented in scientific literature, we relied on cyclists’ perceived comfort feedback. Moreover, for the saddle retraction, the Knee Over Pedal Spindle (K.O.P.S.) method was used [30,31].

Once the bike fitting was finalized, the measurement of the saddle pressures distribution was carried out two weeks later.

### Saddle pressures distribution measurement

Data collection was carried out 48 h after the last training session and each participant was evaluated on own bike installed in a specific bike roller with a magnetic resistance (MagneticDays; Foiano della Chiana, Arezzo, Italy).

The distribution of pressures on the saddle was measured using a Bluetooth device composed of a flexible mat with 68 resistive sensors capable of recording real-time saddle pressures mapping and a transmitter unit (W-Saddle Pro, LetSense Group; Castel Maggiore, Bologna, Italy). The flexible mat was placed on the saddle with its longitudinal midline overlapping the longitudinal midline of the saddle, and the transmitter unit was hung from the rear edge of the saddle.

Before the measurement of pressures distribution on the saddle, each participant was asked to warm-up for 10 min at a self-selected cadence and intensity. Hence, saddle pressures distribution was recorded at three different pedaling intensities (100, 140, 180 W), in a block order from the lowest to the highest workload, with a pedaling cadence of 90 rpm [15,16]. The bike roller provided each participant with real-time feedback on pedaling intensity and cadence in order to maintain those established. Each workload, performed using a sex-neutral saddle (saddle A, Selle Italia S1; Casella d’Asolo, Treviso, Italia), lasted 30 s and a 3-minute rest between workloads was scheduled.

### Parameters of pressure distribution

**Figure 1.**
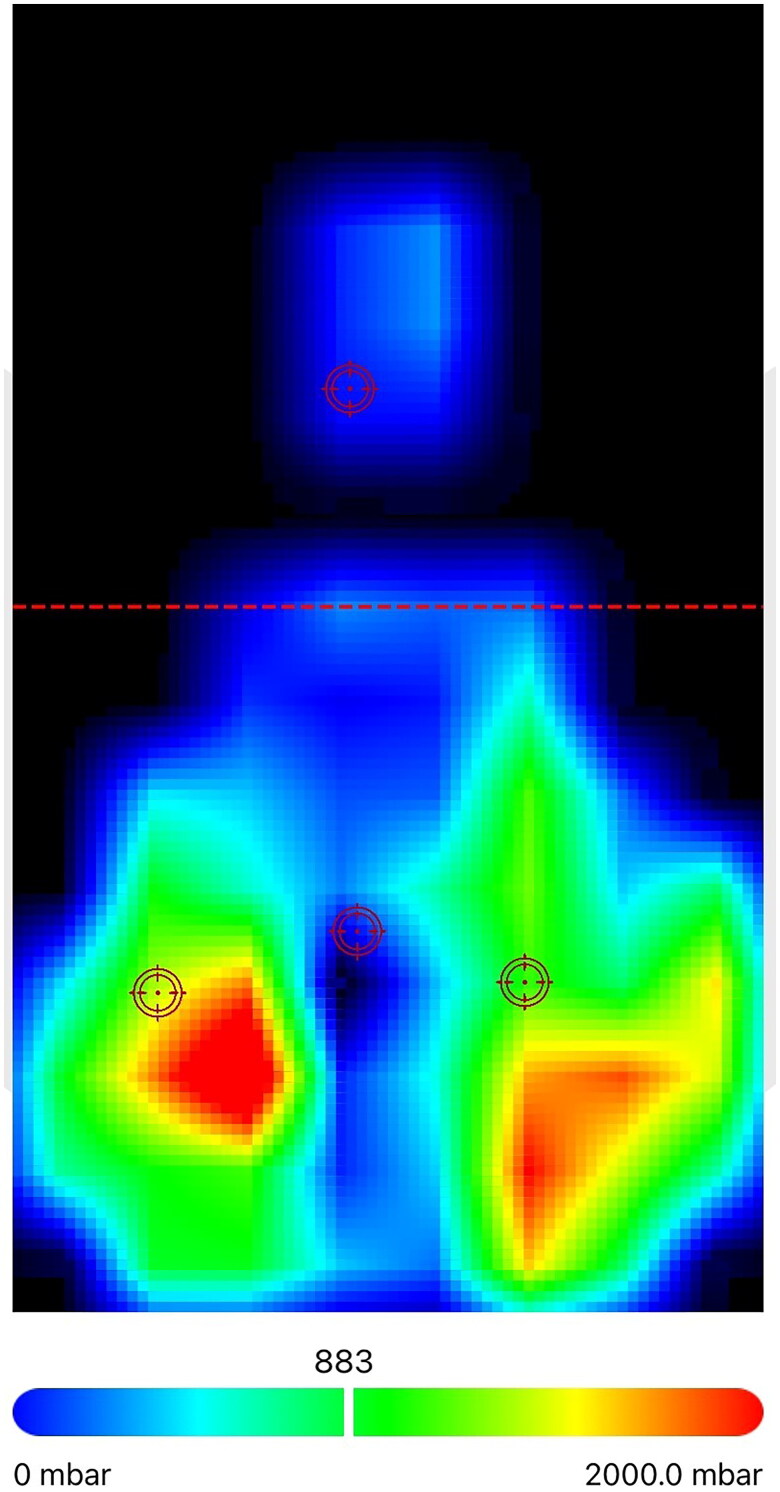
Representation of saddle pressures on a chromatic scale.

The resistive sensors of the flexible mat are divided into three regions: pubic region, left posterior region, and right posterior region. The device provides the following parameters: (a) maximum pubic pressure (mbar); (b) maximum left seat pressure (mbar); (c) maximum right seat pressure (mbar); (d) mean pubic pressure (mbar); (e) mean left seat pressure (mbar); (f) mean right seat pressure (mbar); (g) front pressure (%); (h) back pressure (%); (i) left pressure (%); (j) right pressure (%); (k) ischial distance (mm). The ischial distance was assessed using the same device used for saddle pressures distribution measurement. Figure 1 shows saddle pressures on a chromatic scale.

### Statistical analysis

Data distribution was evaluated using the Shapiro–Wilk’s test. Data were presented as means ± standard deviations.

The unpaired *t*-test was used to assess the difference in the ischial distance between male and female cyclists. The eta-squared was used to assess the effect size for the unpaired *t*-test.

The repeated measures analysis of variance (ANOVA) was used to evaluate any difference in all the saddle pressures parameters among the three trials at different pedaling intensities for both sexes. The partial eta-squared was used to assess the effect size for the ANOVA. In case of significant difference, the Tukey’s *post-hoc* multiple comparisons test between trials was carried out. The Cohen’s *d* was used to assess the effect size for the Tukey’s *post-hoc* multiple comparisons considering the following values for interpretation: 0.2 = small effect; 0.5 = moderate effect; 0.8 = large effect.

Statistical analyses were performed using Statistica software (version 12; StatSoft^®^, TIBCO^®^ Software Inc; Palo Alto, CA, USA) with *p*-value set significant at <0.05.

## Results

Data was normally distributed. Descriptive statistics of all the saddle pressures parameters for each trial are reported in Tables 2 and 3.

**Table 2.** Descriptive statistics of all the saddle pressures parameters for each trial in male.

	Male					
Parameter	Trial at 100 W of pedaling intensity (mean ± SD)	Trial at 140 W of pedaling intensity (mean ± SD)	Trial at 180 W of pedaling intensity (mean ± SD)	*F* value (df = 2.18)	*p* Value	*η*_p_^2^
Maximum pubic pressure (mbar)	514.9 ± 145.6	535.5 ± 208	605.7 ± 241.2	1.71	0.209	0.159
Maximum left seat pressure (mbar)	1010.5 ± 808.1	1055.1 ± 953.9	870.1 ± 551.3	0.45	0.644	0.048
Maximum right seat pressure (mbar)	1256 ± 1854.6	1237.2 ± 1361.8	1191.4 ± 1235	1.68	0.215	0.157
Mean pubic pressure (mbar)	113 ± 68.3	130.9 ± 78.9	145 ± 109.9	0.93	0.411	0.094
Mean left seat pressure (mbar)	225.9 ± 115.4	193.5 ± 133.5	181.4 ± 124	0.85	0.444	0.086
Mean right seat pressure (mbar)	233.4 ± 122.4	222.2 ± 190.9	210 ± 140.1	0.22	0.805	0.024
Front pressure (%)	21.5 ± 16.5	30.9 ± 19.3	38.4 ± 30.7	7.63	0.004	0.459
Back pressure (%)	78.5 ± 16.5	69.1 ± 19.3	61.6 ± 30.7	7.63	0.004	0.459
Left pressure (%)	46.8 ± 9.8	50.6 ± 10.9	48.5 ± 11.8	0.49	0.619	0.052
Right pressure (%)	53.2 ± 9.8	49.4 ± 10.9	51.5 ± 11.8	0.49	0.619	0.052

SD: standard deviation; W: Watt.

**Table 3.** Descriptive statistics of all the saddle pressures parameters for each trial in female.

	Female					
Parameter	Trial at 100 W of pedaling intensity (mean ± SD)	Trial at 140 W of pedaling intensity (mean ± SD)	Trial at 180 W of pedaling intensity (mean ± SD)	*F* value (df = 2.18)	*p* Value	*η*_p_^2^
Maximum pubic pressure (mbar)	266.2 ± 142.3	235.8 ± 144.5	247.6 ± 132.7	0.80	0.465	0.082
Maximum left seat pressure (mbar)	517.3 ± 248.7	603.3 ± 335.3	683.5 ± 401	4.36	0.029	0.326
Maximum right seat pressure (mbar)	569.9 ± 331.2	647.6 ± 370	683.6 ± 324.9	3.38	0.057	0.273
Mean pubic pressure (mbar)	73.8 ± 41.5	57.3 ± 35.1	46 ± 40.5	3.67	0.046	0.289
Mean left seat pressure (mbar)	161.9 ± 82.8	170.9 ± 104.9	166.9 ± 95.2	0.12	0.891	0.013
Mean right seat pressure (mbar)	193.3 ± 118.7	206.9 ± 134	201 ± 129.9	0.20	0.817	0.022
Front pressure (%)	20 ± 14.7	10.7 ± 8.9	10.5 ± 13.5	4.11	0.034	0.313
Back pressure (%)	80 ± 14.7	89.3 ± 8.9	89.5 ± 13.5	4.11	0.034	0.313
Left pressure (%)	47.5 ± 8.7	46.2 ± 10	47.2 ± 9	0.24	0.789	0.026
Right pressure (%)	52.5 ± 8.7	53.8 ± 10	52.8 ± 9	0.24	0.789	0.026

SD: standard deviation; W: Watt.

A significant difference was found in the ischial distance between males and females (*p* = 0.007; *η*^2^ = 0.341).

**Figure 2.**
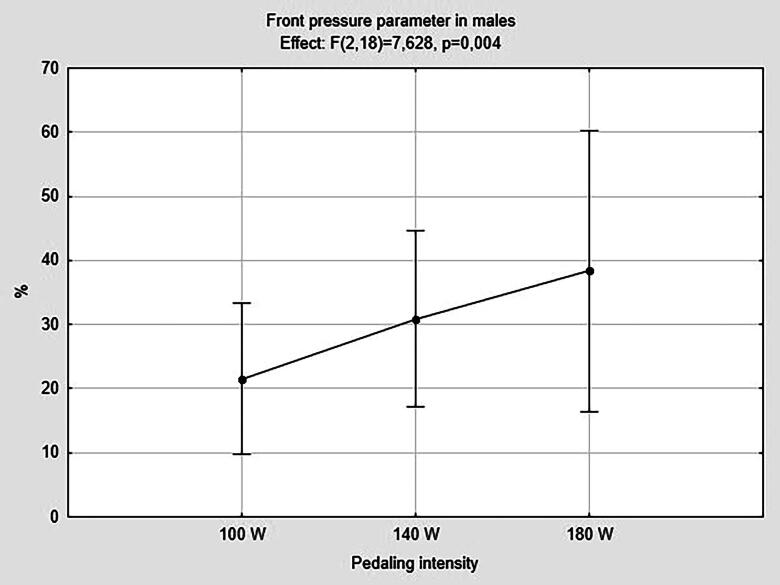
Front pressure parameter in males analyzed using ANOVA repeated measures. *Legend*. Males *n* = 10; W: Watt.

As pedaling intensity increased, males revealed a significant higher pressure in the pubic region (*F*_(2,18)_ = 7.63; *p* = 0.004; *η*_p_^2^ = 0.459), as showed in Figure 2. As a consequence, a significant lower pressure in the posterior region was detected (*F*_(2,18)_ = 7.63; *p* = 0.004; *η*_p_^2^ = 0.459). Post hoc multiple comparisons test revealed a significant increase in the pubic region pressure (*p* = 0.003; *d* = 4.525) and a significant decrease in the posterior region pressure (*p* = 0.003; *d* = 4.525) from 100 to 180 W, as reported in Table 4.

**Table 4.** *Post hoc* analysis of the saddle pressures parameters in male.

	100 W vs. 140 W			140 W vs. 180 W			100 W vs. 180 W		
	Mean diff.	*p* Value	*d*	Mean diff.	*p* Value	*d*	Mean diff.	*p* Value	*d*
Front pressure (%)	9.4	0.104	4.598	7.5	0.222	2.364	16.9	0.003	4.525
Back pressure (%)	−9.4	0.104	4.598	−7.5	0.222	2.364	−16.9	0.003	4.525
W: Watt; d: Cohen’s *d*.									

**Figure 3.**
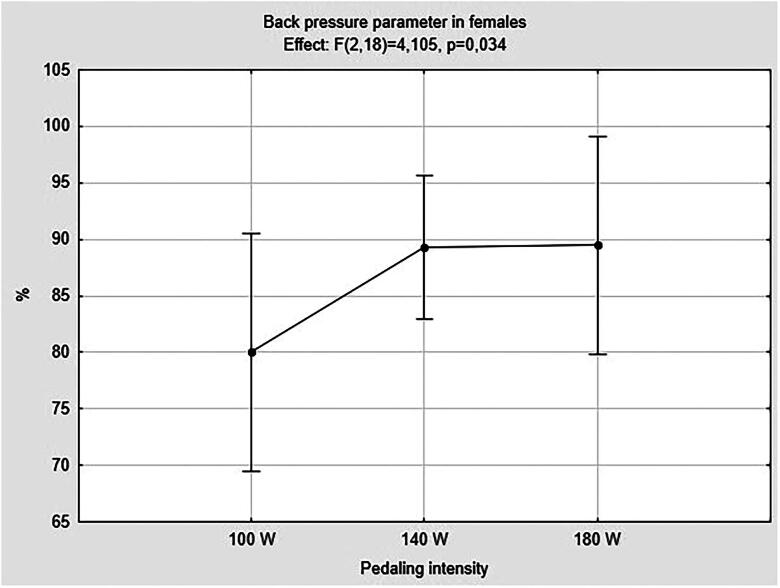
Back pressure parameter in females analyzed using ANOVA repeated measures. Females *n* = 10; W: Watt.

In contrast, females showed a significant higher pressure in the posterior region with increasing pedaling intensity (*F*_(2,18)_ = 4.11; *p* = 0.034; *η*_p_^2^ = 0.313), as showed in Figure 3. As a consequence, a significant lower pressure in the pubic region (*F*_(2,18)_ = 4.11; *p* = 0.034; *η*_p_^2^ = 0.313) was detected. *Post hoc* multiple comparisons test revealed no significant differences in the comparisons among pedaling intensities for these parameters (Table 5).

**Table 5.** *Post hoc* analysis of the saddle pressures parameters in female.

	100 W vs. 140 W			140 W vs. 180 W			100 W vs. 180 W		
	Mean diff.	*p* Value	*d*	Mean diff.	*p* Value	*d*	Mean diff.	*p* Value	*d*
Maximum left seat pressure (mbar)	86	0.302	2.383	80.2	0.350	2.111	166.2	**0.022**	3.711
Mean pubic pressure (mbar)	−16.5	0.272	2.160	−11.3	0.530	2.266	−27.8	**0.038**	3.175
Front pressure (%)	−9.3	0.061	3.359	−0.2	0.999	0.087	−9.5	0.055	3.233
Back pressure (%)	9.3	0.061	3.359	0.2	0.999	0.087	9.5	0.055	3.233

W: Watt; d: Cohen’s d.

A significant difference was found in the mean pubic pressure parameter (*F*_(2,18)_ = 4.11; *p* = 0.046; *η*_p_^2^ = 0.313) with a significant decrease from 100 to 180 W (*p* = 0.038; *d* = 3.175), as shown in Table 5.

Moreover, a significant difference was detected in the maximum left seat pressure parameter (*F*_(2,18)_=4.11; *p* = 0.029; *η*_p_^2^ = 0.313) with a significant increase from 100 to 180 W (*p* = 0.022; *d* = 3.711), as shown in Table 5.

## Discussion

Although different research groups have investigated saddle pressures in cyclists, to the best of our knowledge, there are no studies in the literature that have evaluated pressures on the saddle in young off-road cyclists focusing on sex differences. As a matter of fact, it is known that higher pressures in the pubic region can compress specific biological tissues leading to the onset of chronic genital pathologies [32,33]. Thus, the aim of this study was to investigate any differences on saddle pressures distribution at different pedaling intensity according to sex in young off-road cyclists. Our hypothesis was confirmed because the results showed a significant difference in the distribution of saddle pressures between males and females. In particular, as pedaling intensity increased, higher pressures in the posterior region of the saddle were detected in females compared to males who showed higher pressures in the pubic region of the saddle. In particular, this significant difference was detected from 100 to 180 W. In an opposite way, we detected a significant higher pressure in the posterior region with increasing pedaling intensity in female cyclists and, consequently, a significant lower pressure in the pubic region. In detail, we found an increase in the pressure in the posterior region from 100 to 140 W with similar values between the latter intensity and 180 W. However, *post-hoc* analysis revealed no significant differences among the three different pedaling intensities.

As a matter of fact, it exists a pelvic anatomical difference between males and females, such as the greater width between the ischial tuberosities in females, and this different pelvic geometry can lead to a saddle pressures distribution according to sex [16]. Indeed, in line with the existing literature, we found a significant difference in the ischial distance between male and female cyclists. Previous studies investigated sex-related differences in saddle pressure distributions between male and female cyclists reporting conflicting results [15,16,34]. In a seminal work by Potter et al. (2008) demonstrated that the pelvis-saddle interaction could be an influencing factor on pressures [16] and among the other factors, the pelvic geometry seems to influence saddle pressure distribution [35]. In the latter study it was detected that centers of pressure in the anterior region were significantly higher than females and this finding can be explained by the females’ pelvic bone width [16]. Indeed, females may could move further back on the saddle, which is the widest part of the saddle, in order to accommodate, in a better way, the pelvic bones [16].

In line with the present findings, our preliminary results showed that in a sample of young off-road cyclists, male reported higher pressure in the pubic region as pedaling intensity increased [26]. This could be explained by the variation in pedaling kinematics at different intensities. In fact, Holliday et al. (2023) demonstrated a forward body movement on the bicycle as pedaling intensity increases [36]. These findings are also confirmed in the existing scientific literature [37,38]. In contrast with our results, a study by Holliday et al. (2019), aimed to evaluate any changes in saddle pressures during three different intensities (i.e. 60, 80, and 90% of the maximum heart rate), showed no significant changes in mean pressures in the pubic region in male cyclists [7]. It should be mentioned that the latter study analyzed adult road cyclists. Indeed, among other saddle pressures factors the riding style and saddle design are previously detected [16,39]. Regarding the riding style and according to Sauer et al. (2007), we assume that, with increasing pedaling intensity, male cyclists adopt a cycling posture with the pelvis rotated forward emphasizing the pressures on the anterior pelvic bones [39]. As for the saddle design, although it seems that saddles without a protruding nose reduce pressure in the pubic region, we used the same sex-neutral saddle for all measurements for each participant [18].

A previous review by Partin et al. (2014) reported that, per se, the interaction between cyclists and bike is different according to sex [40] and this factor should be considered during bike fitting for preventing related dysfunctions.

It should be noted that the present findings refer to the time slot in which cyclists sit on the saddle. Indeed, cyclists during a competition or a training session do not sit on the saddle the whole time. However, in a recent review by Vicari et al. (2023) has been analysed the factors influencing saddle pressures finding that sitting time is certainly a factor influencing saddle pressures and related urogenital pathologies.

## Conclusions

The results of this study showed that it exists a difference in saddle pressures distribution between male and female cyclists underlining that the development of saddles properly designed for cyclists according to sex is suitable [18,19]. In particular, it seems that males increase pubic pressure with increasing pedaling intensity while females adopt an opposite strategy.

### Practical implications

These findings can be useful to coaches of young cyclists in order to prevent acute and chronic genital pathologies and, therefore, to improve comfort during pedaling. In fact, we recommend to coaches to tilt the saddle of young male cyclists a couple of degrees with the nose pointing down to reduce pressures in the genital area or to use an increased cushion in the nose [41]. As far as cyclists are concerned, the use of ‘lady’ saddles with designs suited to the anatomical needs of the female perineum is recommended. In addition to set up the saddle, cyclists and technicians should consider other factors that influence saddle pressures [32]. For instance, the maintaining the height of the handlebar lower than the saddle could prevent nerve compression [42,43]. In a similar way, it should be noted that padded cycling shorts increase comfort and can help protect the perineal soft tissue [44]. It should be noted that these suggestions should be carried out periodically thought the bike fitting procedure modifying the cyclist’s setting in relation also to the height and age.

#### Strengths and limitations

The major strength of the study concerns having investigate sex differences in young cyclists, which represents the novelty of the study. Moreover, because of bike set up is a factor that can influence saddle pressure distributions, for this study, all participants were tested on their own bike after a bike fitting in order to avoid any inter-individual factors that could have influenced our measurements. Moreover, all participants used the same sex-neutral saddle.

A limitation of this study concerns the sample size recruited. The post hoc power analysis showed that with a sample of 20 participants, we achieved a power of 65%. Other limitation is that we did not consider the maximum power of each participant, therefore we are not aware of what percentage of the maximum power each participant was pedalling during each trial (i.e. 100, 140, 180 W). Among the limitations it should be mentioned that we did not consider the participants’ pelvic mobility.

### Further studies

Future studies should evaluate saddle pressures during an off-road event and consider analyzing additional factors such as pedaling technique, saddle design, and rider posture.

## Data Availability

The data that support the findings of this study are available from the corresponding author, V.G., upon reasonable request.
